# Why the Prairies Are Treeless

**Published:** 1884-05

**Authors:** 


### WHY THE PRAIRIES ARE TREELESS.

IT is the opinion of Mr. Thomas Meehan that we have nearly reached the solution of the cause of the absence of trees from the prairies. It is not climatic, for timber belts flourish in all the prairie regions. It is not in conditions of soil, for the prairie soil is the most favorable to the germination of seeds of trees as well as of other plants, and artificial plantations are remarkably successful wherever they are made. The real cause is probably to be found in the annual fires which have swept over the prairies from time immemorial, killing the young trees before they can grow large enough to resist the heat. The seeds of the annual plants of the prairie vegetation maturing every year, are shed, and find protection before the fires come; the young trees, on the other hand, bear no seed and leave no resource for a succession after they are burned. This theory is supported by the fact that an abundant growth of trees have set in wherever the fires have been stopped. The fires were made by the aborigines for centuries before the white men came, possibly for the express purpose, Mr. Meehan suggests, of preventing the growth of trees and preserving the buffalo pastures. The question remains how prairies first came to be naked. They probably formed the bottoms of the lakes and marshes that were left after the retreat of the glaciers, and continued wet after the highlands were covered with trees. Man followed the glaciers so closely that he anticipated the trees on those spots, and having learned already in southern latitudes the value of burning them, began before the trees gained a foothold.

